# A Discharge Summary Prompt Reduces Postoperative Radiotherapy Delay in Head and Neck Cancer Patients

**DOI:** 10.1002/hed.70361

**Published:** 2026-06-17

**Authors:** Andreja Radevic, Niketna Vivek, Joaquin Austerlitz, Sindhura Sridhar, Whitney Jin, Robert Sinard, Sarah L. Rohde, Kyle Mannion, Alexander J. Langerman, Eben Rosenthal, Michael C. Topf

**Affiliations:** ^1^ Department of Otolaryngology‐Head and Neck Surgery Vanderbilt University Medical Center Nashville Tennessee USA; ^2^ Texas A&M University College of Medicine Houston Texas USA; ^3^ Baylor College of Medicine Houston Texas USA

**Keywords:** adjuvant radiation, care coordination, head and neck cancer, quality improvement, treatment delay

## Abstract

**Background:**

Delays in postoperative radiotherapy (PORT) beyond 6 weeks are associated with worse outcomes in head and neck cancer (HNC). This study evaluated whether a discharge summary prompt could reduce PORT delay.

**Methods:**

A retrospective review of patients from 2018 to 2023 was used as a pre‐intervention baseline. An EHR‐based discharge summary intervention was implemented in May 2023. Delayed PORT was defined as initiation > 42 days from surgery. Time to PORT was evaluated with Kaplan–Meier and log‐rank testing. Logistic regression identified predictors of delay; Cox modeling evaluated factors associated with time to PORT initiation.

**Results:**

Among 391 patients (260 pre‐intervention, 131 post‐intervention), PORT delay decreased from 74.2% to 46.6% after implementation (*p* < 0.001). The discharge summary prompt was independently associated with lower odds of delay (OR 0.28, 95% CI 0.14–0.56; *p* < 0.001) and faster PORT initiation (HR 2.05, 95% CI 1.43–2.93; *p* < 0.001).

**Conclusions:**

A simple discharge summary prompt may reduce PORT delay in HNC.

## 1 Introduction

As many as two‐thirds of patients who undergo surgery for head and neck cancer (HNC) necessitate multimodal treatment, including adjuvant radiotherapy (RT) or chemoradiotherapy (CRT) [1, 2]. As the incidence of HNC continues to rise [3], awareness and adherence to the National Comprehensive Cancer Network's (NCCN) guidelines [2] for postoperative radiotherapy (PORT) are increasingly important. The significance of these guidelines has been demonstrated in recent studies showing that a time to PORT of greater than 6 weeks is associated with worse overall survival and locoregional control [2, 4, 5, 6, 7, 8]. These findings led both the Commission on Cancer and American Head and Neck Society to implement a quality metric reinforcing the 6‐week window for timely PORT.

Despite the importance of timely PORT, many patients fail to receive PORT within the 6‐week window. A recent meta‐analysis by Duckett et al. reported that up to 49% of patients in the United States fail to receive PORT within this timeframe [9]. Meanwhile, Divi et al. estimate that the optimal rate of timely PORT initiation is approximately 80% given some unavoidable causes of delay [10]. Multiple studies have analyzed risk factors for PORT delay, which include lower socioeconomic status, receipt of PORT at an outside facility, and poor care coordination [11, 12, 13, 14]. While our understanding of the multifactorial nature of delay has improved, there is a scarcity of literature evaluating interventions for mitigating the risk of PORT delay [1, 10]. While a small number of institutions have described quality improvement (QI) interventions aimed at reducing PORT delay rates [1, 10, 15] replicable QI tools are needed if widespread adoption and enduring results are to be achieved.

We previously reported a PORT delay of 73% at our institution [11]. Given these findings, we implemented a simple discharge summary prompt to improve PORT delay. While other studies have examined the implementation of more complex multidisciplinary interventions [7, 10], this investigation utilizes a less resource‐intensive QI intervention which can be easily replicated by other institutions.

## 2 Materials and Methods

### 2.1 Study Design

This study was approved by the Vanderbilt University Medical Center (VUMC) Institutional Review Board (IRB #221228). The study period was from January 1st, 2018–June 1st, 2025. Our cohort was formed from all patients evaluated in the VUMC Head and Neck clinic who received surgery for HNC and required either adjuvant RT or CRT. Patients received PORT at either VUMC or external facilities within Tennessee, Kentucky, Alabama, or Georgia. The primary outcome of this study was PORT delay, defined as time to radiation initiation greater than 42 days. Secondary outcomes included time to PORT initiation (days from surgery to start of RT) and identification of patient, disease, and treatment‐related factors associated with PORT delay.

### 2.2 Patient Selection

We systematically reviewed all surgical cases performed by the faculty head and neck surgeons at our institution and included only patients who underwent definitive oncologic resections for HNC who received PORT. All patients aged 18 years or older requiring adjuvant radiation or chemoradiation therapy were included in this study. Patients who did not receive PORT or were lost to follow up were excluded. The pre‐intervention control group was from January 2018 to May 2023. A QI measure was implemented on May 15, 2023, and intervention group data were collected from May 15th, 2023–June 1st, 2025.

### 2.3 Intervention

An electronic health record (EHR)‐based discharge summary intervention was introduced in May 2023 for patients undergoing HNC surgery (Figure 1). The intervention consisted of a standardized set of prompts embedded within the postoperative discharge summary workflow and was completed by the discharging provider at the time of discharge. The prompts were designed to document key postoperative information relevant to adjuvant RT planning, including whether PORT was anticipated, whether a radiation oncology appointment was made, and whether the patient needed a dental referral. If the discharging provider indicated that a radiation oncology appointment had not been made for a patient anticipated to require PORT, the provider contacted the cancer care coordinator at the time of discharge to facilitate appropriate scheduling. Completion of these questions was intended to improve provider communication and facilitate timely initiation of PORT.

**FIGURE 1:**
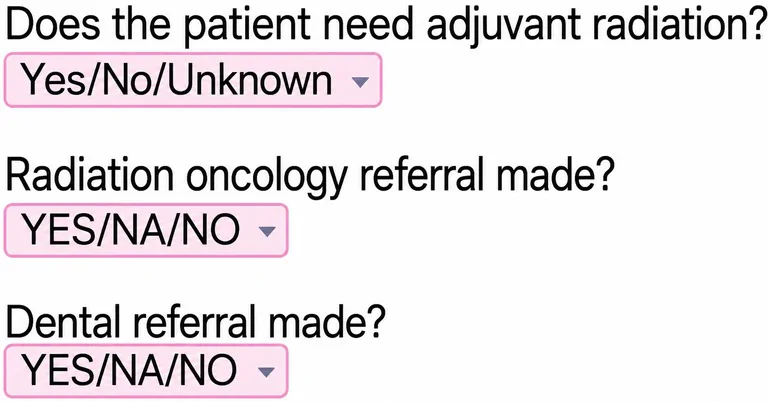
Standardized discharge summary documentation fields from electronic health record. Screenshot of the standardized discharge summary template implemented in May 2023 for patients with head and neck cancer undergoing curative‐intent surgical resection. The template includes three mandatory dropdown fields completed by the provider at postoperative hospital discharge. [Color figure can be viewed at wileyonlinelibrary.com]

### 2.4 Data Collection

De‐identified clinical data was extracted from the VUMC Epic Electronic Health Record (Epic Systems Corporation, Verona, WI, USA) and entered into a REDCap (Research Electronic Data Capture) database. Data abstraction was performed via manual patient chart review by trained research personnel. Data extracted included patient characteristics such as age, sex, race/ethnicity, home address and distance to radiation facility, employment status, education level, insurance status, smoking status, and alcohol use. Tumor characteristics included cancer site, histology, margin status, pathologic tumor (T) and nodal (N) stage, and extranodal extension. Treatment variables included items such as surgery date, surgical and reconstructive procedure, postoperative complications, radiation oncology referral date, need for dental extraction, radiation facility (VUMC or outside radiation facility), and radiation start date. Postoperative complications were defined as documented medical or surgical complications occurring after surgery and before initiation of PORT that were associated with prolonged recovery, extended hospitalization, or additional postoperative management. Postoperative complications requiring return to the operating room were recorded separately.

### 2.5 Statistical Analysis

Baseline characteristics were summarized using descriptive statistics. Differences in the proportion of patients experiencing delayed PORT between the pre‐implementation and discharge summary prompt groups were assessed using the *χ* ^2^ test. Baseline categorical variables between the pre‐ and post‐intervention groups were compared using the *χ* ^2^ test or Fisher's exact test, as appropriate. Continuous variables (e.g., age, distance to radiation facility) were compared using Student's *t*‐test for normally distributed variables and Wilcoxon rank‐sum test for non‐normally distributed variables; normality was assessed using the Shapiro–Wilk test. Univariable logistic regression was conducted to assess associations between individual variables and delayed PORT. Odds ratios (ORs), 95% confidence intervals (CIs), and *p*‐values were estimated for each variable. For time‐to‐event outcomes (days to PORT initiation), univariable Cox proportional hazards regression was performed, with hazard ratios (HRs), 95% CIs, and *p*‐values reported. Statistical significance was defined as *p* < 0.05.

Covariates with significant associations in univariable analyses, as well as clinically relevant variables, were included in multivariable models. Variables were excluded if deemed clinically irrelevant, were collinear, or did not improve model fit. Multivariable logistic regression was used to estimate adjusted ORs for delayed PORT. A multivariable Cox proportional hazards model was constructed for time to initiation of radiation therapy. Because the proportional hazards assumption was violated for pathologic T stage (Schoenfeld residuals test, *p* = 0.012), the final Cox model was stratified by pathologic T stage, allowing for different baseline hazards across T stage groups while estimating common covariate effects.

Model discrimination for logistic regression was assessed using the area under the receiver operating characteristic curve (AUC), and calibration was evaluated with the Hosmer‐Lemeshow goodness‐of‐fit test. For the Cox proportional hazards model, discrimination was assessed using Harrell's concordance index (C‐index), with 95% confidence intervals calculated from the standard error, and overall model fit was assessed using the likelihood ratio test. After stratification by pathologic T stage, the proportional hazards assumption was verified using Schoenfeld residuals with the global test. Multicollinearity among covariates in both models was evaluated using variance inflation factors (VIFs) for the logistic regression model and generalized variance inflation factors (GVIF) for the Cox model, with values below 5 considered acceptable. Internal validation of the Cox model was performed using bootstrap resampling with 200 iterations to assess model stability.

Kaplan–Meier survival curves were generated to estimate time to PORT initiation for included variables. Group differences in time‐to‐event distributions were tested using the log‐rank test. All statistical analyses and data visualization were performed in RStudio (Version 2024.12.1 + 563).

## 3 Results

### 3.1 Cohort Characteristics

Selected baseline demographic, clinical, and treatment characteristics with group comparisons are summarized in Table 1. Full baseline characteristics are provided in Table S1. A total of 391 patients were included, with 260 (66.5%) patients in the pre‐implementation cohort and 131 (33.5%) in the discharge summary prompt cohort. The overall cohort had a mean age of 62.4 years (standard deviation, SD = 12.6) and was predominantly male (*n* = 289, 73.7%) and white (*n* = 348, 89.7%). Most patients were married or partnered (*n* = 252, 64.3%) and had private insurance (*n* = 224, 57.1%). The most common cancer subsite was oral cavity (*n* = 175, 44.6%). Most patients had a pathologic T stage of T3 or higher (*n* = 265, 67.6%) and N stage of N2 or higher (*n* = 220, 56.1%). Most patients received concurrent CRT after surgery (*n* = 224, 57.1%) while 168 (42.9%) patients received postoperative RT alone. Postoperative complications occurred in 56 (14.3%) patients. Most patients received PORT at our institution (*n* = 218, 55.6%). Distance to radiation facility was right‐skewed, with a median distance of 23.9 miles (IQR 10.9–49.4).

**TABLE 1:** Selected baseline characteristics of study cohort by intervention period.

Variable	Pre‐Intervention, *n* (%)	Post‐Intervention, *n* (%)	*p*
Overall	260	131	
Health insurance status			
Medicare	94 (36.6)	43 (32.8)	0.008
Medicaid	3 (1.2)	0 (0.0)	
Private	140 (54.5)	84 (64.1)	
Uninsured	17 (6.6)	0 (0.0)	
VA	3 (1.2)	4 (3.1)	
Marital status			
Single	2 (0.8)	3 (2.5)	0.03
Married/Current partner	181 (73.6)	71 (59.7)	
Separated/Divorced	21 (8.5)	19 (16.0)	
Widowed	42 (17.1)	26 (21.8)	
Race			
White	233 (89.7)	115 (89.6)	0.162
Non‐White	27 (10.3)	13 (9.9)	
Cancer subsite			
Larynx/Hypopharynx	40 (10.2)	34 (13.1)	0.001
Oral cavity	120 (46.2)	55 (42.0)	
Oropharynx	28 (10.8)	30 (22.9)	
Nasal Cavity/Paranasal Sinuses	18 (6.9)	3 (2.3)	
Salivary	16 (6.2)	16 (12.2)	
Skin/Scalp	37 (14.2)	15 (11.5)	
Unknown primary	7 (2.7)	6 (4.6)	
Radiation with or without CRT			
Radiation alone	93 (36.3)	64 (51.2)	0.008
CRT	163 (63.7)	61 (48.8)	
Dental evaluation prior to radiation			
Yes	87 (35.5)	21 (17.2)	< 0.001
No	41 (16.7)	36 (29.5)	
Unable to determine	117 (47.8)	65 (53.3)	
Distance to radiation facility (miles), median (IQR)	24.5 (10.8–49.5)	23.1 (10.4–48.8)	0.44
Postoperative complications			
Yes	36 (14.3)	20 (15.4)	0.91
No	215 (85.7)	110 (84.6)	
Radiation at VUMC			
Yes	111 (53.9)	107 (83.6)	< 0.001
No	95 (46.1)	21 (16.4)	
Pathologic tumor stage			
1	24 (9.4)	25 (19.4)	0.14
2	51 (20.1)	27 (20.9)	
3	66 (26.0)	31 (24.0)	
4	2 (0.8)	0 (0.0)	
4a	96 (27.8)	38 (29.5)	
4b	5 (2.0)	2 (1.6)	
*x*	10 (3.9)	6 (4.7)	
Pathologic nodal stage			
0	67 (27.8)	33 (26.2)	0.002
1	30 (12.4)	42 (33.3)	
2	4 (1.7)	1 (0.8)	
2a	16 (6.6)	9 (7.1)	
2b	26 (10.8)	9 (7.1)	
2c	8 (3.3)	3 (2.4)	
3	5 (2.1)	1 (0.8)	
3a	2 (0.8)	0 (0.0)	
3b	56 (23.2)	10 (15.9)	
*x*	27 (11.2)	8 (6.3)	
Alcohol use			
Current	101 (38.8)	58 (44.3)	0.42
Previous	71 (27.3)	30 (22.9)	
Never	85 (32.7)	43 (32.8)	
Tobacco use			
Current	47 (18.1)	17 (13.0)	0.01
Previous	125 (48.1)	48 (36.6)	
Never	87 (33.5)	66 (50.4)	
Employment status			
Full‐time paid work	61 (23.5)	28 (21.4)	< 0.001
Disability	27 (10.4)	1 (0.8)	
Part‐time paid work	3 (1.2)	2 (1.5)	
Retired	68 (26.2)	7 (5.3)	
Unemployed	16 (6.2)	3 (2.3)	
Other	2 (0.8)	0 (0.0)	
Age at surgery, mean (SD)	62.99 (12.12)	61.37 (13.59)	0.23
Gender			
Female	64 (24.6)	35 (26.7)	0.43
Male	193 (74.2)	96 (73.3)	
Living situation			
Spouse	91 (92.9)	7 (53.8)	< 0.001
Alone	4 (4.1)	2 (15.4)	
Children	0 (0.0)	2 (15.4)	
Friends	1 (1.0)	1 (7.7)	
Parents	2 (2.0)	1 (7.7)	

*Note:* Demographic, clinical, and treatment characteristics of 391 patients with head and neck cancer who underwent curative‐intent surgical resection with a pathologic indication for postoperative radiation therapy (PORT), stratified by pre‐intervention (January 2018–May 2023, *n* = 260) and post‐intervention (May 2023–June 2025, *n* = 131) periods. The intervention was a standardized discharge summary prompt implemented in May 2023. Categorical variables are presented as *n* (%). Continuous variables are presented as mean ± standard deviation or median (interquartile range, IQR), as appropriate. *p*‐values were calculated using *χ* ^2^, independent *t*‐test, or Wilcoxon rank‐sum test. Additional baseline characteristics are provided in Table S1.

Abbreviations: CRT, chemoradiotherapy; IQR, interquartile range; SD, standard deviation; VA, Veteran's Affairs; VUMC, Vanderbilt University Medical Center.

Several baseline characteristics differed significantly between the pre‐implementation and discharge summary prompt cohorts. Notably, the discharge summary prompt cohort had a higher proportion of patients receiving radiation at our institution (83.6% vs. 53.9%, *p* < 0.001) and a lower proportion receiving CRT (48.8% vs. 63.7%, *p* = 0.008). Cancer subsite distribution differed between groups (*p* = 0.001), with the discharge summary prompt cohort having more oropharynx cancers (22.9% vs. 10.8%) and fewer larynx/hypopharynx cancers (4.6% vs. 13.1%). Pathologic N stage distribution also differed (*p* = 0.002), with the discharge summary prompt cohort having more N1 disease (33.3% vs. 12.4%) and less N3b disease (15.9% vs. 23.2%). Marital status (*p* = 0.025) and insurance status (*p* = 0.008) also differed between cohorts; more patients in the pre‐implementation cohort were married (73.6% vs. 59.7%) and fewer were divorced/separated (8.5% vs. 16.0%). More patients in the pre‐implementation cohort were uninsured (6.6% vs. 0%) and fewer had private insurance (54.5% vs. 64.1%).

### 3.2 Primary Outcome: Delayed PORT by Intervention Group

#### 3.2.1 Dichotomous Outcome (Delayed vs. Not Delayed)

Following implementation of the discharge summary intervention, the proportion of patients experiencing delayed PORT initiation (> 42 days) significantly decreased from 74.2% (193/260 patients) to 46.6% (61/131 patients) (*p* < 0.001).

#### 3.2.2 Time‐To‐Event Analysis

Kaplan–Meier log‐rank analysis demonstrated significantly shorter time to PORT initiation in the discharge summary prompt cohort compared to the pre‐implementation cohort (*p* < 0.001) (Figure 2). Median time to PORT was 52 days (95% CI: 50–56; Interquartile Range, IQR: 42–67) in the pre‐implementation group versus 42 days (95% CI: 41–45; IQR: 35–51) in the discharge summary prompt group. The discharge summary prompt cohort also demonstrated reduced variability in treatment timing, with a narrower IQR width (14.5 vs. 25.2 days) and standard deviation (15.8 vs. 44.2 days).

**FIGURE 2:**
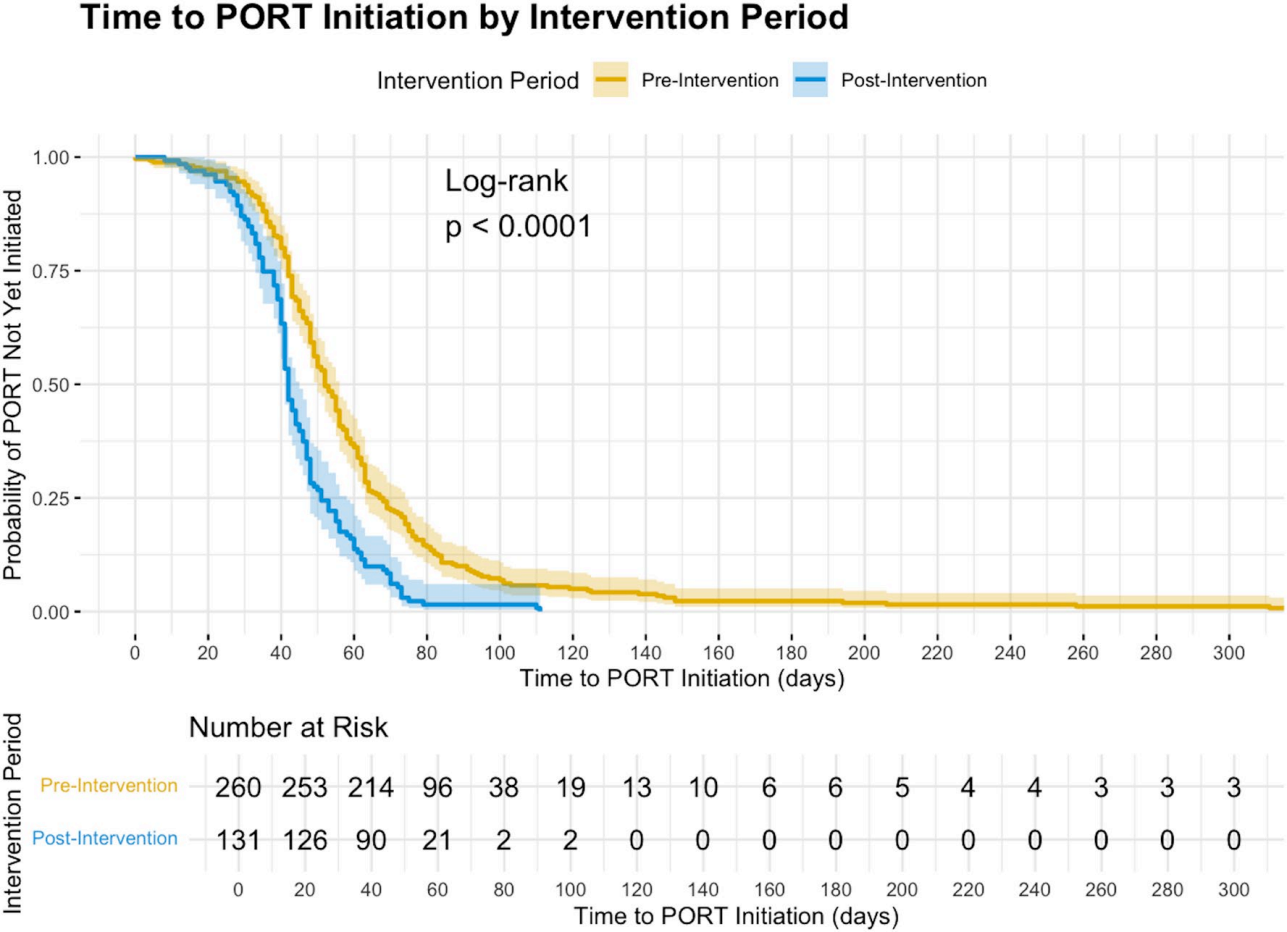
Kaplan–Meier survival curves for time to postoperative radiation therapy (PORT) initiation by intervention period. Kaplan–Meier curves showing time to PORT initiation after curative‐intent surgical resection for head and neck cancer, stratified by intervention period. Shaded regions represent 95% confidence intervals. Time to PORT initiation was shorter in the post‐intervention period following implementation of standardized discharge summary documentation (log‐rank *p* < 0.001). Numbers at risk are shown below the curves. [Color figure can be viewed at wileyonlinelibrary.com]

#### 3.2.3 Multivariable Logistic Regression

On multivariable logistic regression analysis (Table 2), patients in the discharge summary prompt cohort had significantly lower odds of PORT delay (OR = 0.28, 95% CI: 0.14–0.56, *p* < 0.001) when controlling for race, sex, health insurance status, marital status, cancer subsite, concurrent CRT vs. RT alone, dental evaluation, distance to radiation facility, postoperative complications, radiation location, and pathologic stage. Increased odds of delay were also observed in patients with T2 (OR = 4.19, 95% CI: 1.48–12.59, *p* = 0.008), T4a (OR = 3.43, 95% CI: 1.17–10.44, *p* = 0.03), and N3b (OR = 2.96, 95% CI: 1.16–7.90, *p* = 0.03) disease. Interestingly, patients who received radiation alone had increased odds of delay compared to those who received concurrent chemoradiation (OR = 2.92, 95% CI: 1.48–5.98, *p* = 0.003). Univariable logistic regression results are provided in Table S2.

**TABLE 2:** Multivariable logistic regression analysis for delayed postoperative radiation therapy (PORT) initiation.

Variable	Multivariable odds ratio (95% CI)	*p*
Intervention (Ref = Pre‐Intervention)		
Post‐Intervention	0.28 (0.14–0.56)	< 0.001^a^
Health insurance status (Ref = Medicare)		
Medicaid	0.23 (0.007–7.31)	0.35
Private	1.13 (0.59–2.18)	0.71
Uninsured	12.46 (1.50–329.72)	0.051
VA	5.56 (0.35–161.77)	0.24
Marital status (Ref = Single)		
Married/Current‐Partner	1.47 (0.66–3.26)	0.34
Separated/divorced	2.21 (0.71–7.18)	0.18
Widowed	1.22 (0.30–5.31)	0.78
Race (Ref = White)		
Non‐White	0.64 (0.23–1.75)	0.38
Cancer subsite (Ref = Oral Cavity)		
Larynx/Hypopharynx	0.54 (0.19–1.57)	0.26
Oropharynx	2.15 (0.69–7.00)	0.19
Nasal Cavity/Paranasal sinuses	0.69 (0.18–2.78)	0.60
Salivary	1.26 (0.40–4.11)	0.69
Skin/Scalp	1.93 (0.69–5.70)	0.22
Unknown primary	1.64 (0.22–12.30)	0.61
Radiation with or without CRT (Ref = CRT)		
Radiation alone	2.92 (1.48–5.98)	0.003^a^
Dental evaluation prior to radiation (Ref = No)		
Yes	1.68 (0.68–4.21)	0.27
Distance to radiation facility (miles)	1.002 (0.99–1.01)	0.18
Postoperative complications (Ref = No)		
Yes	2.47 (1.006–6.52)	0.056
Radiation at VUMC (Ref = Yes)		
No	1.65 (0.83–3.31)	0.16
Pathologic tumor stage (Ref = Stage 1)		
2	4.19 (1.48–12.59)	0.008^a^
3	2.07 (0.68–6.50)	0.20
4^b^	—	—
4a	3.43 (1.17–10.44)	0.03^a^
4b	6.00 (0.039–11.75)	0.72
*x*	3.69 (0.51–37.32)	0.22
Pathologic nodal stage (Ref = Stage 0)		
1	1.34 (0.53–3.45)	0.54
2	0.73 (0.041–21.11)	0.83
2a	2.20 (0.60–9.13)	0.25
2b	1.26 (0.40–4.09)	0.68
2c	7.99 (0.13–5.50)	0.81
3^b^	—	—
3a^b^	—	—
3b	2.96 (1.16–7.90)	0.03^a^
*x*	1.06 (0.32–3.56)	0.93

*Note:* Odds ratios (OR) and 95% confidence intervals (CIs) for predictors of delayed PORT initiation (> 42 days from surgery) after curative‐intent surgery for head and neck cancer. ORs > 1 indicate increased odds of delay; ORs < 1 indicate decreased odds. Additional univariable logistic regression results are provided in Table S2.

—, not included in the multivariable logistic regression model because of clinical irrelevance, collinearity, or worsened of model fit; see Methods.

Abbreviations: CI, confidence interval; CRT, chemoradiotherapy; Ref, reference; TORS, transoral robotic surgery; VA, Veteran's Affairs; VUMC, Vanderbilt University Medical Center.

^a^ *p* < 0.05 was considered statistically significant.

^b^ Predictor variables with unstable logistic regression results were excluded from this table.

#### 3.2.4 Multivariable Cox Regression

In the adjusted multivariable Cox proportional hazards model (Table 3), patients discharged after implementation of the discharge summary prompt experienced significantly shorter time to PORT initiation compared to patients discharged before implementation (HR = 2.05, 95% CI: 1.43–2.93, *p* < 0.001). Uninsured patients (HR = 0.46, 95% CI: 0.22–0.96, *p* = 0.04) and those with government insurance (HR = 0.22, 95% CI: 0.05–0.87, *p* = 0.03) had longer times to PORT initiation, whereas no significant initiation delays were observed for patients with Medicaid or private insurance. Additionally, receiving RT only (vs. concurrent CRT) (HR = 0.65, 95% CI: 0.47–0.90, *p* = 0.01) was associated with delayed PORT initiation. Postoperative complications also lengthened the time to PORT start (HR = 0.51, 95% CI: 0.33–0.79, *p* = 0.002). In contrast, race, marital status, cancer subsite, dental evaluation, pathologic nodal staging, radiation facility, and distance to the radiation facility did not show statistically significant associations with time to PORT initiation in the adjusted Cox proportional hazards model. Univariable Cox regression results are provided in Table S3.

**TABLE 3:** Multivariable Cox proportional hazards regression analysis of factors associated with time to postoperative radiation therapy (PORT) initiation.

Variable	Multivariable hazard ratio (95% CI)	*p*
Intervention (Ref = Pre‐Intervention)		
Post‐Intervention	2.05 (1.43–2.93)	< 0.001^a^
Health insurance status (Ref = Medicare)		
Medicaid	2.87 (0.61–13.52)	0.18
Private	0.92 (0.68–1.26)	0.60
Uninsured	0.46 (0.22–0.96)	0.04^a^
VA	0.22 (0.05–0.87)	0.03^a^
Marital status (Ref = Single)		
Married/Current‐Partner	1.11 (0.34–3.56)	0.87
Separated/Divorced	0.86 (0.25–2.91)	0.80
Widowed	1.20 (0.36–4.02)	0.77
Race (Ref = White)		
Non‐White	0.96 (0.60–1.54)	0.86
Cancer subsite (Ref = Oral Cavity)		
Larynx/Hypopharynx	0.94 (0.57–1.55)	0.81
Oropharynx	1.06 (0.61–1.84)	0.85
Nasal Cavity/Paranasal Sinuses	1.09 (0.55–2.16)	0.81
Salivary	1.32 (0.76–2.30)	0.33
Skin/Scalp	1.26 (0.78–2.04)	0.34
Unknown primary	1.79 (0.66–4.89)	0.26
Radiation with or without CRT (Ref = CRT)		
Radiation alone	0.65 (0.47–0.90)	0.01^a^
Dental evaluation prior to radiation (Ref = No)		
Yes	0.73 (0.48–1.10)	0.13
Distance to radiation facility (miles)	0.9995 (0.998–1.001)	0.59
Postoperative complications (Ref = No)		
Yes	0.51 (0.33–0.79)	0.002^a^
Radiation at VUMC (Ref = Yes)		
No	0.84 (0.60–1.18)	0.31
Pathologic tumor stage^b^ (Ref = Stage 1)		
2	—	—
3	—	—
4	—	—
4a	—	—
4b	—	—
*x*	—	—
Pathologic nodal stage (Ref = Stage 0)		
1	0.91 (0.57–1.46)	0.70
2	0.48 (0.12–1.96)	0.30
2a	0.98 (0.54–1.78)	0.94
2b	0.85 (0.50–1.46)	0.57
2c	1.90 (0.69–5.25)	0.22
3	1.14 (0.22–5.94)	0.88
3a	1.35 (0.11–16.70)	0.81
3b	0.82 (0.53–1.27)	0.37
*x*	1.09 (0.62–1.94)	0.76

Abbreviations: CI, confidence interval; Ref, reference; VA, Veteran's Affairs; CRT, chemoradiotherapy; VUMC, Vanderbilt University Medical Center.

*Note:* Hazard ratios (HR) and 95% confidence intervals (CIs) for factors associated with time to PORT initiation following curative‐intent surgery for head and neck cancer. The multivariable model was stratified by pathologic tumor stage and adjusted for prespecified covariates. HRs > 1 indicate shorter time to PORT initiation; HRs < 1 indicate longer time to initiation. Additional univariable Cox regression results are provided in Table S3.

—, not included in the multivariable Cox model because clinical irrelevance, collinearity, or worsened of model fit; see Methods.

^a^ *p* < 0.05 was considered statistically significant.

^b^ Pathologic T stage not included in the multivariable Cox model as it was used as a stratification variable to address violation of the proportional hazards assumption; see Methods.

## 4 Discussion

Timely PORT initiation after head and neck cancer surgery remains challenging despite national quality metrics targeting treatment within 6 weeks [2]. In this single‐institution cohort (*n* = 391), delayed PORT initiation (> 42 days) decreased from 74.2% to 46.6% after implementation of a simple EHR‐based discharge summary prompt (adjusted OR 0.28, 95% CI: 0.14–0.56, *p* < 0.001). Time to PORT improved (median 52 to 42 days; log‐rank *p* < 0.001), with faster initiation in adjusted time‐to‐event analysis (HR 2.05, 95% CI: 1.43–2.93, *p* < 0.001). Although baseline characteristics differed between pre‐ and post‐intervention groups (including radiation facility and receipt of adjuvant chemotherapy), the association between the intervention period and timeliness persisted after multivariable adjustment. These findings suggest that a simple, standardized discharge summary handoff may be a practical, low‐resource tool to decrease PORT delay.

Prior work suggests PORT delay is multifactorial, reflecting patient‐level barriers (including social determinants of health), as well as system‐level failures in care coordination, communication, and fragmentation of surgical and radiation oncology workflows [4, 8, 9, 11, 12, 13, 14, 16, 17]. Multilevel interventions have improved timeliness, often by adding patient navigators and redesigning referral and tracking processes. Graboyes et al. reported improved care coordination and high guideline‐adherent PORT initiation (86% overall) using the NDURE model, which incorporates patient education, travel support, PORT care plans, and referral tracking and follow‐up [1]. Similarly, Divi et al. implemented a multidisciplinary workflow intervention targeting dental and radiation oncology workflows, decreasing delayed PORT initiation from 38% to 27% [10]. However, these approaches required substantial resource investment and may be difficult to replicate in smaller academic or community hospital settings. In contrast, our intervention was embedded within routine discharge documentation and did not require new personnel or major workflow redesign. Notably, less resource‐intensive efforts, such as an additional preoperative visit to address treatment‐related questions, have been associated with reduced odds of delay (adjusted OR ~ 0.30, *p* = 0.026) [15]. These findings suggest that simple, targeted interventions may offer substantial benefits while remaining financially and logistically accessible.

Poor care coordination following surgery is a major contributor to PORT delay. In a recent prospective study, poor care coordination was the primary reason for delay in 41.3% of patients who did not achieve timely PORT, underscoring the importance of referral and scheduling workflows. Dental clearance is another persistent barrier, with prior studies linking delays in dental referral and clearance to radiation delays [8, 10, 18]. In contrast, dental evaluation and extraction were not independently associated with time to PORT in our adjusted Cox model, suggesting this relationship may vary across practice settings. Disease complexity may also influence timeliness; in our adjusted logistic model, higher pathologic stage (T2/T4a and N3b) was independently associated with delay. System‐level fragmentation has also been associated with delayed adjuvant therapy, including barriers related to insurance insecurity and neighborhood‐level disadvantage [14, 19]. In our cohort, insurance type remained associated with timeliness in the adjusted Cox model, and uninsured status showed a borderline association with delay in the adjusted logistic model (adjusted OR 12.45, *p* = 0.051). Together, these findings suggest that while standardized discharge documentation may improve care coordination, additional targeted support is likely needed for patients at high risk of delay [20].

One notable strength of this intervention is its intentional simplicity. The three‐question, “yes/no/not applicable” format was integrated into the existing discharge documentation workflow without requiring major process redesign, additional infrastructure, or new personnel, distinguishing it from previously described interventions to reduce PORT delay. Prior interventions have often relied on complex workflow redesign, patient navigation, and multidisciplinary tracking, whereas a simple discharge summary prompt may be easier to implement, sustain, and scale, particularly in resource‐limited settings. Outside of head and neck oncology, standardized discharge summary templates have been associated with improved documentation quality [21], more timely completion [22, 23], greater provider satisfaction [24, 25], and better communication with outpatient providers [21, 23]. This broader literature suggests that standardized, workflow‐embedded documentation can improve downstream care coordination. Our study extends this principle to head and neck oncology, where a simple discharge prompt was associated with substantially improved timeliness of PORT initiation.

The findings of this study should be interpreted in the context of its limitations. First, the pre‐post design limits causal inference, and other time‐related changes such as staffing, scheduling capacity, provider awareness, or referral practices may have influenced PORT timeliness. However, the improvement in PORT timeliness was observed across multiple analytic approaches and remained significant after multivariable adjustment. Second, some baseline characteristics differed between cohorts (including radiation facility and postoperative treatment modality), raising the possibility of confounding. A higher proportion of patients in the discharge summary prompt cohort received RT within our cancer center, which may have improved timeliness through more efficient scheduling and direct communication between multidisciplinary teams. The reasons for this shift were not captured in our dataset but may reflect evolving referral patterns and the increasing presence of regional oncology services. Because the distance to the RT facility was similar between cohorts, this difference was unlikely to be explained by geographic proximity alone. Although we adjusted for radiation facility and distance to radiation facility in the multivariable logistic and Cox models, residual confounding from concurrent system‐level changes in referral patterns or network expansion cannot be excluded. Third, the multivariable Cox model relied on complete‐case analysis (*n* = 256) and missing covariate data could introduce selection bias. However, the primary outcome analysis included the full cohort and showed a similarly large, directionally consistent improvement, making it less likely that the findings were explained solely by the reduced Cox sample. Finally, we did not directly measure completion of the discharge prompt or downstream referral completion, limiting our ability to determine which components drove improvement. Nevertheless, the intervention was intentionally simple and embedded in routine discharge workflow, supporting its real‐world feasibility and scalability.

Improving the timeliness of PORT is a clear opportunity to raise adherence to an established quality metric, and our findings suggest that an EHR‐based discharge prompt can shorten the postoperative interval to adjuvant radiation treatment. For patients, a more reliable transition from surgery to adjuvant therapy may reduce uncertainty after discharge and potentially improve oncologic outcomes. For clinicians, this work supports standardizing the discharge care transition as a key step in the PORT workflow. The discharge prompt could further be converted into an automated order set that incorporates radiation oncology and dental referrals, and communicates confirmation once consults are scheduled. Furthermore, improved PORT timeliness aligns with national benchmarks and may avert downstream costs associated with recurrence and treatment intensification, supporting adoption of a scalable EHR workflow [26, 27, 28]. Moving forward, combining this low‐resource intervention with targeted solutions for higher‐risk patients offers a practical pathway to reducing residual delays and improving equitable access to timely adjuvant therapy.

## 5 Conclusions

A simple EHR‐based discharge summary prompt was associated with a significant reduction in delayed PORT after head and neck cancer surgery and shifted treatment timing toward the 6‐week benchmark. Although PORT delay remains multifactorial, standardizing the discharge handoff represents a practical quality improvement strategy in head and neck cancer care.

## Author Contributions

**Andreja Radevic:** conceptualization, data curation, investigation, methodology, visualization, writing – original draft, writing – review and editing, formal analysis. **Niketna Vivek:** conceptualization, data curation, investigation, methodology, visualization, writing – original draft, writing – review and editing, formal analysis. **Joaquin Austerlitz:** data curation, investigation, methodology, writing – original draft, writing – review and editing. **Sindhura Sridhar:** data curation, investigation, methodology, writing – original draft, writing – review and editing. **Whitney Jin:** data curation, investigation, methodology, writing – original draft, writing – review and editing. **Robert Sinard:** writing – original draft, writing – review and editing, investigation. **Sarah L. Rohde:** writing – original draft, writing – review and editing, investigation. **Kyle Mannion:** writing – original draft, writing – review and editing, investigation. **Alexander J. Langerman:** writing – original draft, writing – review and editing, investigation. **Eben Rosenthal:** writing – original draft, writing – review and editing, investigation. **Michael C. Topf:** conceptualization, data curation, methodology, funding acquisition, investigation, supervision, visualization, writing – original draft, writing – review and editing, resources.

## Funding

This work was supported by a National Cancer Institute (NCI) K08 Career Development Award—5K08CA293255‐02.

## Disclosure

No material from other sources was reproduced in this study. All figures and data are original.

## Ethics Statement

This study was conducted with institutional review board approval (IRB #221228). The study was conducted in accordance with the ethical standard of the Declaration of Helsinki.

## Conflicts of Interest

The authors declare no conflicts of interest.

## Supporting information


**Table S1:** Full Baseline Characteristics of the Study Cohort by Intervention Period. Demographic, clinical, and treatment characteristics of patients with head and neck cancer who underwent curative‐intent surgical resection with a pathologic indication for postoperative radiation therapy (PORT), stratified by intervention period. The intervention was a standardized discharge summary prompt implemented in May 2023. Categorical variables are presented as *n* (%). Continuous variables are presented mean ± standard deviation, or median (interquartile range, IQR), as appropriate. *p*‐values were calculated using χ^2^, independent *t*‐test, or Wilcoxon rank‐sum test. Abbreviations: CI, confidence interval; Ref, reference; VA, Veteran's Affairs; CRT, chemoradiotherapy; VUMC, Vanderbilt University Medical Center; TORS, transoral robotic surgery; FF, free flap; SCC, squamous cell carcinoma; BCC, basal cell carcinoma; ENE, extranodal extension; HPV, human papillomavirus.

**Table S2:** Univariable Logistic Regression Analysis for Delayed Postoperative Radiation Therapy (PORT) Initiation. Odds ratios (ORs) and 95% confidence intervals (CIs) for candidate predictors of delayed PORT initiation (> 42 days from surgery) after curative‐intent surgery for head and neck cancer. Each predictor was assessed separately in a univariable logistic regression model. ORs > 1 indicate increased odds of delay; ORs < 1 indicate decreased odds. Abbreviations: CI, confidence interval; Ref, reference; VA, Veteran's Affairs; CRT, chemoradiotherapy; VUMC, Vanderbilt University Medical Center; TORS, transoral robotic surgery; FF, free flap; SCC, squamous cell carcinoma; BCC, basal cell carcinoma; ENE, extranodal extension; HPV, human papillomavirus.

**Table S3:** Univariable Cox Regression Analysis for Time Postoperative Radiation Therapy (PORT) Initiation. Hazard ratios (HRs) and 95% confidence intervals (CIs) are shown for candidate predictors of time to PORT initiation after curative‐intent surgery for head and neck cancer. Each predictor was assessed separately in a univariable Cox proportional hazards regression model. HRs > 1 indicate shorter time to PORT initiation; HRs < 1 indicate longer time to initiation. Abbreviations: CI, confidence interval; Ref, reference; VA, Veteran's Affairs; CRT, chemoradiotherapy; VUMC, Vanderbilt University Medical Center; TORS, transoral robotic surgery; FF, free flap; SCC, squamous cell carcinoma; BCC, basal cell carcinoma; ENE, extranodal extension; HPV, human papillomavirus.

## Data Availability

The data that support the findings of this study are available from the corresponding author upon reasonable request.

## References

[1] E. M. Graboyes , K. R. Sterba , H. Li , et al., “Development and Evaluation of a Navigation‐Based, Multilevel Intervention to Improve the Delivery of Timely, Guideline‐Adherent Adjuvant Therapy for Patients With Head and Neck Cancer,” JCO Oncology Practice 17 (2021): 1512–1523, 10.1200/OP. PMC8791819 33689399

[2] R. I. Haddad , W. L. Hicks , Y. J. Hitchcock , et al., “NCCN Guidelines Version 4.2025 Head and Neck Cancers Continue NCCN Guidelines Panel Disclosures,” 2025, https://www.nccn.org/home/member.

[3] R. L. Siegel , T. B. Kratzer , A. N. Giaquinto , H. Sung , and A. Jemal , “Cancer Statistics, 2025,” CA: a Cancer Journal for Clinicians 75, no. 1 (2025): 10–45, 10.3322/caac.21871. PMC11745215 39817679

[4] E. A. Noyes , C. A. Burks , A. R. Larson , and D. G. Deschler , “An Equity‐Based Narrative Review of Barriers to Timely Postoperative Radiation Therapy for Patients With Head and Neck Squamous Cell Carcinoma,” Laryngoscope Investigative Otolaryngology 6, no. 6 (2021): 1358–1366, 10.1002/lio2.692. PMC8665479 34938875

[5] N. Villemure‐Poliquin , R. Fu , K. Gaebe , et al., “Delayed Postoperative Radiotherapy in Head & Neck Cancers—A Systematic Review and Meta‐Analysis,” Laryngoscope 135, no. 5 (2025): 1563–1570, 10.1002/lary.31990. PMC11980969 39744810

[6] A. S. Ho , S. Kim , M. Tighiouart , et al., “Quantitative Survival Impact of Composite Treatment Delays in Head and Neck Cancer,” Cancer 124, no. 15 (2018): 3154–3162, 10.1002/cncr.31533. PMC6097917 29742280

[7] E. M. Graboyes , E. Garrett‐Mayer , M. A. Ellis , et al., “Effect of Time to Initiation of Postoperative Radiation Therapy on Survival in Surgically Managed Head and Neck Cancer,” Cancer 123, no. 24 (2017): 4841–4850, 10.1002/cncr.30939. PMC5759768 28841234

[8] T. A. Janz , J. Kim , E. G. Hill , et al., “Association of Care Processes With Timely, Equitable Postoperative Radiotherapy in Patients With Surgically Treated Head and Neck Squamous Cell Carcinoma,” JAMA Otolaryngology Head & Neck Surgery 144, no. 12 (2018): 1105–1114, 10.1001/jamaoto. PMC6472989 30347012

[9] K. A. Duckett , M. F. Kassir , S. A. Nguyen , et al., “Delays Starting Postoperative Radiotherapy Among Head and Neck Cancer Patients: A Systematic Review and Meta‐Analysis,” Otolaryngology and Head and Neck Surgery 170, no. 2 (2024): 320–334, 10.1002/ohn.538. PMC10840985 37731255

[10] V. Divi , M. M. Chen , W. Hara , et al., “Reducing the Time From Surgery to Adjuvant Radiation Therapy: An Institutional Quality Improvement Project,” Otolaryngology and Head and Neck Surgery 159, no. 1 (2018): 158–165, 10.1177/0194599818768254. 29631478

[11] N. Vivek , R. Sharma , K. Prasad , et al., “Impact of Postoperative Radiation Therapy Delay and Treatment Facility Location on Survival in Head and Neck Cancer Patients,” Head & Neck 47, no. 7 (2025): 1807–1815, 10.1002/hed.28092. PMC12146822 40485334

[12] E. M. Graboyes , C. H. Halbert , H. Halbert , et al., “Barriers to the Delivery of Timely, Guideline‐Adherent Adjuvant Therapy Among Patients With Head and Neck Cancer CORRESPONDING AUTHOR Barriers to the Delivery of Timely, Guideline‐Adherent Adjuvant Therapy Among Patients With Head and Neck Cancer,” JCO Oncology Practice 16 (2020): 1417–1432, 10.1200/OP.20. PMC7735037 32853120

[13] K. A. Duckett , M. F. Kassir , S. A. Nguyen , et al., “Factors Associated With Head and Neck Cancer Postoperative Radiotherapy Delays: A Systematic Review and Meta‐Analysis,” Otolaryngology and Head and Neck Surgery 171, no. 5 (2024): 1265–1282, 10.1002/ohn.835. PMC11499046 38842034

[14] E. M. Graboyes , J. L. Cagle , S. Ramadan , et al., “Neighborhood‐Level Disadvantage and Delayed Adjuvant Therapy in Head and Neck Cancer,” JAMA Otolaryngology Head & Neck Surgery 150, no. 6 (2024): 472–482, 10.1001/jamaoto.2024.0424. PMC11046410 38662392

[15] A. A. Bon‐Nieves , N. C. Wang , S. F. Wagoner , et al., “Impact of Presurgical Second‐Touch Visits on Delays in Initiating Adjuvant Radiation Therapy,” Otolaryngology and Head and Neck Surgery 172, no. 6 (2025): 1980–1987, 10.1002/ohn.1194. 40211658

[16] J. Pang , F. Faraji , E. Risa , L. K. Mell , J. J. Houlton , and J. A. Califano , “High Rates of Postoperative Radiotherapy Delay in Head and Neck Cancer Before and After Medicaid Expansion,” Head & Neck 43, no. 9 (2021): 2672–2684, 10.1002/hed.26736. 33998088

[17] M. M. Chen , U. C. Megwalu , J. Liew , D. Sirjani , E. L. Rosenthal , and V. Divi , “Regionalization of Head and Neck Cancer Surgery May Fragment Care and Impact Overall Survival,” Laryngoscope 129, no. 6 (2019): 1413–1419, 10.1002/lary.27440. 30152007

[18] R. Arch , S. J. Ma , E. Gogineni , et al., “Delays in Radiation Therapy for Head and Neck Cancer: The Role of Pre‐Radiation Dental Clearance,” Otolaryngology–Head and Neck Surgery 174 (2025): 430–437, 10.1002/ohn.70096. 41451693

[19] D. A. Levy , H. Li , K. R. Sterba , et al., “Development and Validation of Nomograms for Predicting Delayed Postoperative Radiotherapy Initiation in Head and Neck Squamous Cell Carcinoma,” JAMA Otolaryngology Head & Neck Surgery 146, no. 5 (2020): 455–464, 10.1001/jamaoto.2020.0222. PMC7118672 32239201

[20] L. A. Gharzai , J. Bromfield , M. Kwan , et al., “Implementation Mapping to Identify Strategies to Increase Timely Postoperative Radiotherapy Initiation for Head/Neck Cancer,” Otolaryngology–Head and Neck Surgery 173, no. 1 (2025): 288–298, 10.1002/ohn.1268. PMC12207378 40304500

[21] S. L. Banker , D. Lakhaney , B. S. Hooe , T. A. McCann , C. Kostacos , and M. Lane , “A Quality Improvement Approach to Improving Discharge Documentation,” Pediatric Quality and Safety 7, no. 1 (2022): e428. 10.1097/pq9.0000000000000428 PMC10997293 38586219

[22] A. Larrow , A. Chong , T. Robison , et al., “A Quality Improvement Initiative to Improve Discharge Timeliness and Documentation,” Pediatric Quality and Safety 6, no. 4 (2021): e440. 10.1097/pq9.0000000000000440 PMC8322483 34345753

[23] S. F. Murphy , L. Lenihan , F. Orefuwa , G. Colohan , I. Hynes , and C. G. Collins , “Electronic Discharge Summary and Prescription: Improving Communication Between Hospital and Primary Care,” Irish Journal of Medical Science 186, no. 2 (2017): 455–459, 10.1007/s11845-016-1397-7. 26809947

[24] S. M. Dean , A. Gilmore‐Bykovskyi , J. Buchanan , B. Ehlenfeldt , and A. J. H. Kind , “Design and Hospitalwide Implementation of a Standardized Discharge Summary in an Electronic Health Record,” Joint Commission Journal on Quality and Patient Safety 42, no. 12 (2016): 555, 10.1016/S1553-7250(16)30107-6. PMC5367268 28334559

[25] K. A. Lynch , S. W. Baron , S. Rikin , et al., “Improving Resident Hospital Discharge Communication by Changing Electronic Health Record Templates to Enhance Primary Care Provider Satisfaction,” Quality Management in Healthcare 33, no. 2 (2024): 112–120. 10.1097/QMH.0000000000000417 37482635

[26] K. Sun , J. Y. Tan , P. J. Thomson , and S. W. Choi , “Influence of Time Between Surgery and Adjuvant Radiotherapy on Prognosis for Patients With Head and Neck Squamous Cell Carcinoma: A Systematic Review,” Head Neck 45, no. 8 (2023): 2108–2119, 10.1002/hed.27401. 37194205

[27] M. D. Fisher , A. W. Fernandes , T. O. Olufade , P. J. Miller , M. S. Walker , and M. Fenton , “Patient Characteristics and Costs in Recurrent or Refractory Head and Neck Cancer: Retrospective Analysis of a Community Oncology Database,” Clinical Therapeutics 40, no. 4 (2018): 562–573, 10.1016/j.clinthera.2018.02.010. 29530457

[28] M. Van Agthoven , B. M. Van Ineveld , M. F. De Boer , et al., “The Costs of Head and Neck Oncology: Primary Tumours, Recurrent Tumours and Long‐Term Follow‐Up,” European Journal of Cancer 37, no. 17 (2001): 2204–2211. 10.1016/s0959-8049(01)00292-1 11677108

