# Supplementary material for: A Discharge Summary Prompt Reduces Postoperative Radiotherapy Delay in Head and Neck Cancer Patients
Source: Head Neck. 2026 Jun 17;48(12):3309–19. doi: 10.1002/hed.70361 (PMC13472147; doi:10.1002/hed.70361)
Supplement: Supplementary file 1 [file HED-48-3309-s001.docx]

| **Variable** | **Pre-Intervention, n (%)** | **Post-Intervention, n (%)** | **p-value** |
| --- | --- | --- | --- |
| **Overall** | 260 | 131 |  |
| **Health Insurance Status** |  |  | 0.008 |
| Medicare | 94 (36.6) | 43 (32.8) |  |
| Medicaid | 3 (1.2) | 0 (0.0) |  |
| Private | 140 (54.5) | 84 (64.1) |  |
| Uninsured | 17 (6.6) | 0 (0.0) |  |
| VA | 3 (1.2) | 4 (3.1) |  |
| **Marital Status** |  |  | 0.03 |
| Single | 2 (0.8) | 3 (2.5) |  |
| Married/Current Partner | 181 (73.6) | 71 (59.7) |  |
| Separated/Divorced | 21 (8.5) | 19 (16.0) |  |
| Widowed | 42 (17.1) | 26 (21.8) |  |
| **Race** |  |  | 0.162 |
| White | 233 (89.7) | 115 (89.6) |  |
| Non-White | 27 (10.3) | 13 (9.9) |  |
| **Cancer Subsite** |  |  | 0.001 |
| Larynx/Hypopharynx | 40 (10.2) | 34 (13.1) |  |
| Oral Cavity | 120 (46.2) | 55 (42.0) |  |
| Oropharynx | 28 (10.8) | 30 (22.9) |  |
| Nasal Cavity/Paranasal Sinuses | 18 (6.9) | 3 (2.3) |  |
| Salivary | 16 (6.2) | 16 (12.2) |  |
| Skin/Scalp | 37 (14.2) | 15 (11.5) |  |
| Unknown primary | 7 (2.7) | 6 (4.6) |  |
| **Radiation with or without CRT** |  |  | 0.008 |
| Radiation alone | 93 (36.3) | 64 (51.2) |  |
| CRT | 163 (63.7) | 61 (48.8) |  |
| **Dental Evaluation Prior to Radiation** |  |  | <0.001 |
| Yes | 87 (35.5) | 21 (17.2) |  |
| No | 41 (16.7) | 36 (29.5) |  |
| Unable to Determine | 117 (47.8) | 65 (53.3) |  |
| **Distance to Radiation Facility (miles), mean (SD)** | 54.58 (124.38) | 40.38 (61.44) | 0.22 |
| **Postoperative Complications** |  |  | 0.91 |
| Yes | 36 (14.3) | 20 (15.4) |  |
| No | 215 (85.7) | 110 (84.6) |  |
| **Radiation at VUMC** |  |  | <0.001 |
| Yes | 111 (53.9) | 107 (83.6) |  |
| No | 95 (46.1) | 21 (16.4) |  |
| **Pathologic Tumor Stage** |  |  | 0.14 |
| 1 | 24 (9.4) | 25 (19.4) |  |
| 2 | 51 (20.1) | 27 (20.9) |  |
| 3 | 66 (26.0) | 31 (24.0) |  |
| 4 | 2 (0.8) | 0 (0.0) |  |
| 4a | 96 (27.8) | 38 (29.5) |  |
| 4b | 5 (2.0) | 2 (1.6) |  |
| x | 10 (3.9) | 6 (4.7) |  |
| **Pathologic Nodal Stage** |  |  | 0.002 |
| 0 | 67 (27.8) | 33 (26.2) |  |
| 1 | 30 (12.4) | 42 (33.3) |  |
| 2 | 4 (1.7) | 1 (0.8) |  |
| 2a | 16 (6.6) | 9 (7.1) |  |
| 2b | 26 (10.8) | 9 (7.1) |  |
| 2c | 8 (3.3) | 3 (2.4) |  |
| 3 | 5 (2.1) | 1 (0.8) |  |
| 3a | 2 (0.8) | 0 (0.0) |  |
| 3b | 56 (23.2) | 10 (15.9) |  |
| x | 27 (11.2) | 8 (6.3) |  |
| **Alcohol Use** |  |  | 0.42 |
| Current | 101 (38.8) | 58 (44.3) |  |
| Previous | 71 (27.3) | 30 (22.9) |  |
| Never | 85 (32.7) | 43 (32.8) |  |
| **Tobacco Use** |  |  | 0.01 |
| Current | 47 (18.1) | 17 (13.0) |  |
| Previous | 125 (48.1) | 48 (36.6) |  |
| Never | 87 (33.5) | 66 (50.4) |  |
| **Employment Status** |  |  | <0.001 |
| Full-Time Paid Work | 61 (23.5) | 28 (21.4) |  |
| Disability | 27 (10.4) | 1 (0.8) |  |
| Part-Time Paid Work | 3 (1.2) | 2 (1.5) |  |
| Retired | 68 (26.2) | 7 (5.3) |  |
| Unemployed | 16 (6.2) | 3 (2.3) |  |
| Other | 2 (0.8) | 0 (0.0) |  |
| **Age at Surgery, mean (SD)** | 62.99 (12.12) | 61.37 (13.59) | 0.23 |
| **Gender** |  |  | 0.43 |
| Female | 64 (24.6) | 35 (26.7) |  |
| Male | 193 (74.2) | 96 (73.3) |  |
| **Living Situation** |  |  | <0.001 |
| Spouse | 91 (92.9) | 7 (53.8) |  |
| Alone | 4 (4.1) | 2 (15.4) |  |
| Children | 0 (0.0) | 2 (15.4) |  |
| Friends | 1 (1.0) | 1 (7.7) |  |
| Parents | 2 (2.0) | 1 (7.7) |  |
| **Dental Extraction Prior to Radiation** |  |  | <0.001 |
| Yes | 55 (36.7) | 9 (10.5) |  |
| No | 95 (63.3) | 77 (89.5) |  |
| **Required Dental Extraction** |  |  | <0.001 |
| Yes | 55 (36.4) | 9 (9.8) |  |
| No | 96 (63.6) | 83 (90.2) |  |
| **Previous History of Head & Neck Cancer** |  |  | 0.07 |
| Yes | 52 (21.6) | 16 (13.0) |  |
| No | 189 (78.4) | 107 (87.0) |  |
| **Days to Radiation Oncology Referral (from surgery date), mean (SD)** | 21.27 (54.50) | 13.21 (19.87) | 0.13 |
| **Postoperative Complications Requiring Surgery** |  |  | 0.001 |
| Yes | 44 (16.9) | 12 (9.2) |  |
| No | 197 (75.8) | 118 (90.1) |  |
| **Days From Surgery to Postoperative Complications Requiring Surgery, mean (SD)** | 25.61 (40.11) | 16.15 (13.75) | 0.41 |
| **Ablative Surgery** |  |  |  |
| External Skin Resection | 35 (13.5) | 7 (5.3) | 0.02 |
| Glossectomy | 15 (5.8) | 23 (17.6) | <0.001 |
| Maxillectomy | 5 (1.9) | 6 (4.6) | 0.24 |
| Mandibulectomy | 9 (3.5) | 3 (2.3) | 0.75 |
| Neck dissection | 187 (71.9) | 102 (77.9) | 0.25 |
| Other | 86 (33.1) | 16 (12.2) | <0.001 |
| Parotidectomy | 53 (20.4) | 22 (16.8) | 0.48 |
| Partial Glossectomy | 59 (22.7) | 28 (21.4) | 0.87 |
| Partial Laryngectomy | 1 (0.4) | 0 (0.0) | 1.00 |
| Partial Mandibulectomy | 22 (8.5) | 10 (7.6) | 0.93 |
| Partial Maxillectomy | 13 (5.0) | 3 (2.3) | 0.31 |
| Radical Tonsillectomy | 10 (3.8) | 5 (3.8) | 1.00 |
| TORS | 20 (7.7) | 13 (9.9) | 0.58 |
| Total Laryngectomy | 28 (10.8) | 5 (3.8) | 0.03 |
| **Reconstructive Surgery** |  |  |  |
| Allograft | 1 (0.4) | 0 (0.0) | 1.00 |
| Fasciocutaneous FF | 90 (34.6) | 32 (24.4) | 0.05 |
| None | 81 (31.2) | 52 (41.2) | 0.06 |
| Osseocutaneous FF | 30 (11.5) | 16 (12.2) | 0.98 |
| Regional Flap | 40 (15.4) | 29 (22.1) | 0.13 |
| Skin Graft | 16 (6.2) | 7 (5.3) | 0.93 |
| **Histology** |  |  | 0.03 |
| p16+ SCC Mucosa | 21 (8.1) | 26 (19.8) |  |
| Acinic Cell Carcinoma | 0 (0.0) | 2 (1.5) |  |
| Adenoid Cystic Carcinoma | 9 (3.5) | 5 (3.8) |  |
| BCC skin | 1 (0.4) | 1 (0.8) |  |
| Mucoepidermoid Carcinoma | 1 (0.4) | 1 (0.8) |  |
| Other | 24 (9.2) | 12 (9.2) |  |
| Other Salivary Cancer | 8 (3.1) | 4 (3.1) |  |
| p16- SCC Mucosa | 171 (65.8) | 65 (49.6) |  |
| SCC Skin | 22 (8.5) | 14 (10.7) |  |
| **ENE** |  |  | 0.38 |
| Yes | 102 (39.5) | 45 (34.4) |  |
| No | 156 (60.5) | 86 (65.6) |  |
| **Final Margins** |  |  | 0.30 |
| Positive | 51 (19.9) | 18 (14.2) |  |
| Not Applicable (No Primary Tumor) | 9 (3.5) | 3 (2.4) |  |
| Negative | 196 (76.6) | 106 (83.5) |  |
| **HPV Status (Ref = Negative)** |  |  | 0.32 |
| Positive | 22 (75.9) | 25 (86.2) |  |
| Unknown | 2 (6.9) | 0 (0.0) |  |
| Negative | 5 (17.2) | 4 (13.8) |  |

**Supplemental Table 1. Full Baseline Characteristics of the Study Cohort by Intervention Period:** Demographic, clinical, and treatment characteristics of patients with head and neck cancer who underwent curative-intent surgical resection with a pathologic indication for postoperative radiation therapy (PORT), stratified by intervention period. The intervention was a standardized discharge summary prompt implemented in May 2023. Categorical variables are presented as n (%). Continuous variables are presented mean ± standard deviation, or median (interquartile range, IQR), as appropriate. P-values were calculated using χ², independent t-test, or Wilcoxon rank-sum test. Abbreviations: CI, confidence interval; Ref, reference; VA, Veteran’s Affairs; CRT, chemoradiotherapy; VUMC, Vanderbilt University Medical Center; TORS, transoral robotic surgery; FF, free flap; SCC, squamous cell carcinoma; BCC, basal cell carcinoma; ENE, extranodal extension; HPV, human papillomavirus.
