# Supplementary material for: A Discharge Summary Prompt Reduces Postoperative Radiotherapy Delay in Head and Neck Cancer Patients
Source: Head Neck. 2026 Jun 17;48(12):3309–19. doi: 10.1002/hed.70361 (PMC13472147; doi:10.1002/hed.70361)
Supplement: Supplementary file 2 [file HED-48-3309-s002.docx]

| **Variable** | **Univariable Odds Ratio (95% CI)** | **p-value** |
| --- | --- | --- |
| **Intervention (Ref = Pre-Intervention)** |  |  |
| Post-Intervention | 0.31 (0.20 - 0.48) | <0.001* |
| **Health Insurance Status (Ref = Medicare)** |  |  |
| Medicaid | 1.15 (0.11 - 25.10) | 0.91 |
| Private | 0.99 (0.64 - 1.55) | 0.98 |
| Uninsured | 9.20 (1.79- 168.0) | 0.03* |
| VA | 3.45 (0.57 - 66.10) | 0.26 |
| **Marital Status (Ref= Single)** |  |  |
| Married/Current -Partner | 0.45 (0.02 - 3.10) | 0.48 |
| Separated/divorced | 0.66 (0.03 - 5.10) | 0.72 |
| Widowed | 0.36 (0.02 - 2.57) | 0.37 |
| **Race (Ref = White)** |  |  |
| Non-White | 0.80 (0.41 - 1.59) | 0.51 |
| **Cancer Subsite (Ref = Oral Cavity)** |  |  |
| Larynx/Hypopharynx | 1.03 (0.50 - 2.20) | 0.94 |
| Oropharynx | 0.70 (0.38 - 1.30) | 0.31 |
| Nasal Cavity/Paranasal sinuses | 0.55 (0.22 - 1.38) | 0.47 |
| Salivary | 0.83 (0.38 - 1.85) | 0.63 |
| Skin/Scalp | 1.12 (0.58 - 2.22) | 0.75 |
| Unknown primary | 0.79 (0.25 - 2.72) | 0.70 |
| **Radiation with or without CRT (Ref = CRT)** |  |  |
| Radiation alone | 1.43 (0.93 - 2.22) | 0.11 |
| **Dental Evaluation Prior to Radiation (Ref = No)** |  |  |
| Yes | 2.13 (1.13 - 4.03) | 0.02* |
| **Distance to Radiation Facility (miles)** | 1.00 (0.99 - 1.01) | 0.25 |
| **Postoperative Complications (Ref = No)** |  |  |
| Yes | 1.58 (0.86 - 3.06) | 0.16 |
| **Radiation at VUMC (Ref = Yes)** |  |  |
| No | 1.95 (1.21 - 3.21) | 0.007* |
| **Pathologic Tumor Stage (Ref = Stage 1)** |  |  |
| 2 | 2.90 (1.40 - 6.15) | 0.005* |
| 3 | 2.35 (1.17 - 4.80) | 0.02* |
| 4^†^ | - | - |
| 4a | 3.53 (1.80 - 7.06) | <0.001* |
| 4b | 1.93 (0.39 - 10.70) | 0.42 |
| x | 6.28 (1.76 - 30.10) | 0.009* |
| **Pathologic Nodal Stage (Ref = Stage 0)** |  |  |
| 1 | 0.72 (0.39 - 1.32) | 0.28 |
| 2 | 0.96 (0.15 - 7.53) | 0.96 |
| 2a | 2.02 (0.78 - 5.96) | 0.17 |
| 2b | 1.39 (0.63 - 3.26) | 0.43 |
| 2c | 1.12 (0.32 - 4.50) | 0.87 |
| 3 ^†^ | - | - |
| 3a ^†^ | - | - |
| 3b | 1.92 (1.00 - 3.75) | 0.052 |
| x | 0.96 (0.44 - 2.14) | 0.92 |
| **Alcohol Use (Ref = Never)** |  |  |
| Current | 0.85 (0.52 - 1.39) | 0.52 |
| Previous | 0.85 (0.49 - 1.46) | 0.55 |
| **Tobacco Use (Ref = Never)** |  |  |
| Current | 1.64 (0.90 - 1.06) | 0.11 |
| Previous | 2.02 (1.28 - 3.22) | 0.003* |
| **Employment Status (Ref = Full-Time Paid Work)** |  |  |
| Disability | 1.94 (0.64 - 1.82) | 0.78 |
| Part-Time Paid Work | 0.97 (0.15 - 7.66) | 0.98 |
| Retired | 1.38 (0.73 - 2.64) | 0.33 |
| Unemployed | 2.43 (0.81 - 9.07) | 0.14 |
| Other | 0.65 (0.03 - 16.70) | 0.76 |
| **Age at Surgery** | 1.00 (0.99 - 1.02) | 0.57 |
| **Gender** |  |  |
| Female | 1.09 (0.68 - 1.76) | 0.74 |
| Male | 0.92 (0.57 - 1.48) | 0.74 |
| **Living Situation (Ref = Spouse)** |  |  |
| Alone | 1.02 (0.19 - 7.60) | 0.99 |
| Children^†^ | - | - |
| Friends | 0.51 (0.02 - 13.10) | 0.64 |
| Parents | 1.24 (0.012 - 2.74) | 0.27 |
| **Dental Extraction Prior to Radiation (Ref = no)** |  |  |
| Yes | 2.44 (1.27 - 5.00) | 0.01* |
| **Required Dental Extraction (Ref = No)** |  |  |
| Yes | 2.58 (1.34 - 5.26) | 0.006* |
| **Previous History of Head & Neck Cancer (Ref = No)** |  |  |
| Yes | 1.11 (0.64 - 1.96) | 0.72 |
| **Days to Radiation Oncology Referral (from surgery date)** | 1.02 (1.01 - 1.03) | <0.001* |
| **Postoperative Complications Requiring Surgery (Ref = No)** |  |  |
| Yes | 1.64 (0.89 - 3.17) | 0.13 |
| **Days From surgery to Postoperative Complications requiring Surgery** | 1.04 (1.00 - 1.09) | 0.09 |
| **Ablative Surgery (Ref = Not Performed)** |  |  |
| External Skin Resection | 1.24 (0.64 - 2.56) | 0.53 |
| Glossectomy | 1.38 (0.68 - 2.99) | 0.39 |
| Maxillectomy | 0.65 (0.19 - 2.28) | 0.48 |
| Mandibulectomy | 2.80 (0.72 - 18.40) | 0.78 |
| Neck dissection | 1.12 (0.70 - 1.79) | 0.63 |
| Other | 0.90 (0.72 - 1.13) | 0.36 |
| Parotidectomy | 1.03 (0.61 - 1.77) | 0.90 |
| Partial Glossectomy | 0.81 (0.50 - 1.33) | 0.40 |
| Partial Laryngectomy^†^ | - | - |
| Partial Mandibulectomy | 1.22 (0.57 - 2.77) | 0.62 |
| Partial Maxillectomy | 2.44 (0.77 - 10.80) | 0.17 |
| Radical Tonsillectomy | 1.52 (0.51 - 5.58) | 0.48 |
| TORS | 0.72 (0.35 - 1.51) | 0.37 |
| Total Laryngectomy | 0.95 (0.46 - 2.05) | 0.89 |
| **Reconstructive Surgery (Ref = Not Performed)** |  |  |
| Allograft^†^ | - | - |
| Fasciocutaneous FF | 1.46 (0.93 - 2.33) | 0.11 |
| None | 0.85 (0.55 - 1.31) | 0.46 |
| Osseocutaneous FF | 1.63 (0.84 - 3.39) | 0.17 |
| Regional Flap | 0.66 (0.39 - 1.12) | 0.12 |
| Skin Graft | 0.69 (0.30 - 1.67) | 0.40 |
| **Histology (Ref = p16-positive SCC Mucosa)** |  |  |
| Acinic Cell Carcinoma^†^ | - | - |
| Adenoid Cystic Carcinoma | 1.58 (0.47 - 5.82) | 0.47 |
| BCC skin | 0.88 (0.03 - 23.10) | 0.93 |
| Mucoepidermoid Carcinoma^†^ | - | - |
| Other | 1.38 (0.58 - 3.38) | 0.47 |
| Other Salivary Cancer | 2.64 (0.69 - 13.00) | 0.18 |
| p16-negative SCC Mucosa | 1.75 (0.92 - 3.30) | 0.08 |
| SCC Skin | 2.64 (1.05 - 7.06) | 0.045* |
| **ENE (Ref = No)** |  |  |
| Yes | 1.51 (0.98 - 2.36) | 0.06 |
| **Final Margins (Ref = Negative)** |  |  |
| Positive | 0.70 (0.41 - 1.19) | 0.18 |
| Not Applicable (No Primary Tumor) | 0.70 (0.22 - 2.43) | 0.56 |
| **HPV Status (Ref = Negative)** |  |  |
| Positive | 0.35 (0.05 - 1.65) | 0.22 |
| Unknown | 0.29 (0.008 - 9.45) | 0.44 |

**Supplemental Table 2. Univariable Logistic Regression Analysis for Delayed Postoperative Radiation Therapy (PORT) Initiation.** Odds ratios (ORs) and 95% confidence intervals (CIs) for candidate predictors of delayed PORT initiation (>42 days from surgery) after curative-intent surgery for head and neck cancer. Each predictor was assessed separately in a univariable logistic regression model. ORs >1 indicate increased odds of delay; ORs <1 indicate decreased odds. Abbreviations: CI, confidence interval; Ref, reference; VA, Veteran’s Affairs; CRT, chemoradiotherapy; VUMC, Vanderbilt University Medical Center; TORS, transoral robotic surgery; FF, free flap; SCC, squamous cell carcinoma; BCC, basal cell carcinoma; ENE, extranodal extension; HPV, human papillomavirus.
*p<0.05 was considered statistically significant.
†Predictor variables with unstable logistic regression results were excluded from this table.
–, not included because of clinical irrelevance, collinearity, or worsening of model fit; see Methods.
