# Supplementary material for: A Discharge Summary Prompt Reduces Postoperative Radiotherapy Delay in Head and Neck Cancer Patients
Source: Head Neck. 2026 Jun 17;48(12):3309–19. doi: 10.1002/hed.70361 (PMC13472147; doi:10.1002/hed.70361)
Supplement: Supplementary file 3 [file HED-48-3309-s003.docx]

| **Variable** | **Univariable Hazard Ratio (95% CI)** | **p-value** |
| --- | --- | --- |
| **Intervention (Ref = Pre-Intervention)** |  |  |
| Post-Intervention | 2.01 (1.62 - 2.50) | <0.001* |
| **Health Insurance Status (Ref = Medicare)** |  |  |
| Medicaid | 1.36 (0.43 - 24.26) | 0.60 |
| Private | 0.97 (0.78 - 1.20) | 0.76 |
| Uninsured | 0.41 (0.25- 1.70) | <0.001* |
| VA | 0.57 (0.27 - 1.22) | 0.92 |
| **Marital Status (Ref= Single)** |  |  |
| Married/Current -Partner | 1.17 (0.48 - 2.83) | 0.73 |
| Separated/Divorced | 1.04 (0.41 - 2.63) | 0.94 |
| Widowed | 0.91 (0.37 - 2.26) | 0.84 |
| **Race (Ref = White)** |  |  |
| Non-White | 0.90 (0.65 - 1.26) | 0.55 |
| **Cancer Subsite (Ref = Oral Cavity)** |  |  |
| Larynx/Hypopharynx | 0.79 (0.56 - 1.12) | 0.19 |
| Oropharynx | 1.46 (1.08 - 1.97) | 0.01* |
| Nasal Cavity/Paranasal Sinuses | 1.14 (0.72 - 1.80) | 0.57 |
| Salivary | 1.75 (1.20 - 2.57) | 0.004* |
| Skin/Scalp | 1.08 (0.79 - 1.48) | 0.61 |
| Unknown primary | 1.13 (0.64 - 1.98) | 0.68 |
| **Radiation with or without CRT (Ref = CRT)** |  |  |
| Radiation alone | 0.87 (0.71 - 1.06) | 0.17 |
| **Dental Evaluation Prior to Radiation (Ref = No)** |  |  |
| Yes | 0.61 (0.46 - 0.83) | 0.001* |
| **Distance to Radiation Facility (miles)** | 1.00 (0.99 - 1.01) | 0.24 |
| **Postoperative Complications (Ref = No)** |  |  |
| Yes | 0.65 (0.48 - 0.87) | 0.004* |
| **Radiation at VUMC (Ref = Yes)** |  |  |
| No | 0.75 (0.60 - 0.94) | 0.01* |
| **Pathologic Tumor Stage** ^‡^ **(Ref = Stage 1)** |  |  |
| 2 | 0.69 (0.48 - 0.99) | 0.04* |
| 3 | 0.66 (0.47 - 0.93) | 0.02* |
| 4 | 0.46 (0.11 - 1.18) | 0.28 |
| 4a | 0.57 (0.41 - 0.79) | <0.001* |
| 4b | 0.60 (0.27 - 1.32) | 0.20 |
| x | 0.52 (0.29 - 0.91) | 0.02* |
| **Pathologic Nodal Stage (Ref = Stage 0)** |  |  |
| 1 | 1.36 (1.002 - 1.85) | 0.049* |
| 2 | 0.82 (0.33 - 2.02) | 0.67 |
| 2a | 0.94 (0.60 - 1.46) | 0.78 |
| 2b | 0.78 (0.53 - 1.16) | 0.22 |
| 2c | 1.26 (0.68 - 2.36) | 0.46 |
| 3 | 0.83 (0.36 - 1.89) | 0.65 |
| 3a | 1.19 (0.29 - 4.85) | 0.81 |
| 3b | 0.90 (0.67 - 1.22) | 0.49 |
| x | 0.98 (0.67 - 1.44) | 0.92 |
| **Alcohol Use (Ref = Never)** |  |  |
| Current | 0.83 (0.72 - 1.15) | 0.41 |
| Previous | 0.95 (0.73 - 1.24) | 0.73 |
| **Tobacco Use (Ref = Never)** |  |  |
| Current | 0.83 (0.62 - 1.12) | 0.23 |
| Previous | 0.91 (0.73 - 1.13) | 0.39 |
| **Employment Status (Ref = Full-Time Paid Work)** |  |  |
| Disability | 0.64 (0.42 - 0.98) | 0.04* |
| Part-Time Paid Work | 0.96 (0.39 - 2.37) | 0.93 |
| Retired | 0.89 (0.65 - 1.21) | 0.46 |
| Unemployed | 0.61 (0.37 - 1.007) | 0.054 |
| Other | 0.73 (0.18 - 2.97) | 0.66 |
| **Age at Surgery** | 0.99 (0.991 - 1.008) | 0.92 |
| **Gender** |  |  |
| Female | 0.82 (0.26 - 2.58) | 0.73 |
| Male | 1.04 (0.33 - 3.25) | 0.94 |
| **Living Situation (Ref = Spouse)** |  |  |
| Alone | 1.49 (0.64 - 3.43) | 0.35 |
| Children | 3.56 (0.85 - 14.95) | 0.083 |
| Friends | 3.16 (0.76 - 13.07) | 0.11 |
| Parents | 0.78 (0.24 - 2.52) | 0.68 |
| **Dental Extraction Prior to Radiation (Ref = no)** |  |  |
| Yes | 0.62 (0.46 - 0.83) | 0.001* |
| **Required Dental Extraction (Ref = No)** |  |  |
| Yes | 0.58 (0.43 - 0.77) | <0.001* |
| **Previous History of Head & Neck Cancer (Ref = No)** |  |  |
| Yes | 0.83 (0.64 - 1.09) | 0.18 |
| **Days to Radiation Oncology Referral (from surgery date)** | 0.99 (0.992 - 0.998) | <0.001* |
| **Postoperative Complications Requiring Surgery (Ref = No)** |  |  |
| Yes | 0.66 (0.49 - 0.87) | 0.004* |
| **Days From Surgery to Postoperative Complications Requiring Surgery** | 0.988 (0.98 - 0.99) | 0.01* |
| **Ablative Surgery (Ref = Not Performed)** |  |  |
| External Skin Resection | 1.00 (0.73 - 1.38) | 0.99 |
| Glossectomy | 0.98 (0.70 - 1.37) | 0.90 |
| Maxillectomy | 0.92 (0.51 - 1.69) | 0.80 |
| Mandibulectomy | 0.92 (0.51 - 1.64) | 0.77 |
| Neck dissection | 1.00 (0.80 - 1.26) | 0.98 |
| Other | 0.90 (0.72 - 1.13) | 0.36 |
| Parotidectomy | 1.25 (0.97 - 1.62) | 0.08 |
| Partial Glossectomy | 1.00 (0.79 - 1.28) | 0.99 |
| Partial Laryngectomy | 1.00 (0.06 - 2.97) | 0.38 |
| Partial Mandibulectomy | 0.83 (0.57 - 1.20) | 0.33 |
| Partial Maxillectomy | 0.73 (0.44 - 1.21) | 0.23 |
| Radical Tonsillectomy | 0.92 (0.55 - 1.54) | 0.75 |
| TORS | 1.07 (0.75 - 1.53) | 0.70 |
| Total Laryngectomy | 0.85 (0.59 - 1.22) | 0.37 |
| **Reconstructive Surgery (Ref = Not Performed)** |  |  |
| Allograft | 3.07 (0.43 - 21.95) | 0.27 |
| Fasciocutaneous FF | 0.81 (0.65 - 1.003) | 0.053 |
| None | 1.24 (1.001 - 1.52) | 0.049* |
| Osseocutaneous FF | 0.77 (0.57 - 1.05) | 0.10 |
| Regional Flap | 0.98 (0.75 - 1.27) | 0.86 |
| Skin Graft | 0.94 (0.62 - 1.44) | 0.79 |
| **Histology (Ref = p16-positive SCC Mucosa)** |  |  |
| Acinic Cell Carcinoma | 2.99 (0.72 - 12.46) | 0.13 |
| Adenoid Cystic Carcinoma | 0.68 (0.37 - 1.22) | 0.19 |
| BCC skin | 0.31 (0.07 - 1.28) | 0.11 |
| Mucoepidermoid Carcinoma | 5.12 (1.23 - 21.33) | 0.03* |
| Other | 0.82 (0.53 - 1.27) | 0.37 |
| Other Salivary Cancer | 0.87 (0.46 - 1.64) | 0.66 |
| p16-negative SCC Mucosa | 0.64 (0.47 - 0.88) | 0.006* |
| SCC Skin | 0.73 (0.47 - 1.13) | 0.15 |
| **ENE (Ref = No)** |  |  |
| Yes | 0.96 (0.78 - 1.18) | 0.70 |
| **Final Margins (Ref = Negative)** |  |  |
| Positive | 1.02 (0.79 - 1.33) | 0.86 |
| Not Applicable (No Primary Tumor) | 1.09 (0.61 - 1.94) | 0.78 |
| **HPV Status (Ref = Negative)** |  |  |
| Positive | 1.02 (0.49 - 2.11) | 0.96 |
| Unknown | 1.10 (0.23 - 5.13) | 0.91 |

**Supplemental Table 3. Univariable Cox Regression Analysis for Time Postoperative Radiation Therapy (PORT) Initiation.** Hazard ratios (HRs) and 95% confidence intervals (CIs) are shown for candidate predictors of time to PORT initiation after curative-intent surgery for head and neck cancer. Each predictor was assessed separately in a univariable Cox proportional hazards regression model. HRs >1 indicate shorter time to PORT initiation; HRs <1 indicate longer time to initiation. Abbreviations: CI, confidence interval; Ref, reference; VA, Veteran’s Affairs; CRT, chemoradiotherapy; VUMC, Vanderbilt University Medical Center; TORS, transoral robotic surgery; FF, free flap; SCC, squamous cell carcinoma; BCC, basal cell carcinoma; ENE, extranodal extension; HPV, human papillomavirus.
*p<0.05 was considered statistically significant.
